# A Cross-Sectional Study of Mental Illnesses Among Infertile Women in Saudi Arabia

**DOI:** 10.7759/cureus.63823

**Published:** 2024-07-04

**Authors:** Munirah Alsahel, Majed Alghamdi

**Affiliations:** 1 Preventive Medicine, Ministry of Health Saudi Arabia, Jeddah, SAU; 2 Preventive Medicine, Joint Program of Preventive Medicine, Jeddah, SAU; 3 Public Health Administration, Saudi Board of Preventive Medicine Program, Ministry of Health Saudi Arabia, Jeddah, SAU

**Keywords:** reproductive age, quality of life, mental health, infertility, dass-21

## Abstract

Background: Infertility is a global issue and carries significant mental health implications. Data on mental health among infertile women in Saudi Arabia are limited.

Objective: This study aimed to assess the prevalence and severity of depression, anxiety, and stress among infertile women in Saudi Arabia.

Methods: This analytical cross-sectional study included women of reproductive age visiting governmental hospitals in Jeddah, Saudi Arabia. The participants were asked to fill out a pre-structured questionnaire, which included the Depression, Anxiety, and Stress Scale (DASS-21) to assess their mental health status. Data analysis was conducted using IBM SPSS Statistics, Version 29.0 (released 2023, IBM Corp., Armonk, NY). The three outcome variables derived from the DASS-21 were categorized into five distinct groups for descriptive purposes.

Results: In this study, infertile women had significantly higher median scores for depression (18), anxiety (15), and stress (20) compared to fertile women who had median scores of 8, 8, and 10 for depression, anxiety, and stress, respectively. The differences between these two groups were statistically significant (p-value < 0.001). Furthermore, employed infertile women reported higher median scores for depression (87), anxiety (84.5), and stress (84.5) compared to unemployed women. In addition, infertile women with a monthly income of 10,001-20,000 SAR had notably higher median scores for depression (89.56), anxiety (90.22), and stress (89.94) compared to other income groups. These differences were statistically significant (p-values < 0.05).

Conclusion: Infertility significantly contributes to mental health issues among women in Saudi Arabia. The findings highlight the need for targeted psychological interventions alongside infertility treatment to enhance the quality of life of these women.

## Introduction

Patients from all over the world are affected by the life crisis of infertility. According to the World Health Organization, infertility is defined as the failure to conceive after 12 months or more of regular, unprotected sexual activity [1]. Worldwide, approximately one in six adults, or 17.5%, struggle with infertility [1]. It has been shown that infertility has an impact on how families and couples interact. It has been proven in the past that anxiety brought on by infertility is linked to depressive symptoms [2].

Utilizing reliable measurement tools is essential to providing accurate assessments of the mental health status of women who are experiencing infertility. The Depression Anxiety Stress Scale 21 (DASS-21) questionnaire is a recent example of such a tool that has gained prominence. The DASS-21 questionnaire, developed to address shortcomings in current emotional measures, assesses levels of depression and anxiety. Its effectiveness depends on its ability to distinguish these two conditions with accuracy [3]. The scale has 21 items in total, evenly distributed into three subscales (stress, anxiety, and depression), each with seven items. Each subsection's overall score can be anywhere between normal and extremely severe.

Numerous studies have been done that show infertile women have a high risk of mental health issues. A study by Fallahzadeh et al. revealed that infertile couples experienced higher levels of depression and anxiety than fertile couples [4]. Another study by Sezgin et al. found that infertile women were more significantly subjected to clinically significant anxiety symptoms than fertile women (31% vs. 17%) [5]. Another study in Taiwan reported that over 40% of 112 women receiving infertility treatment met the diagnostic criteria for one of these common mental disorders, which is significantly higher than the 10-12% reported in the general population. Generalized anxiety disorder was present in 23.2% of those, major depressive disorder in 17.0%, and dysthymic disorder in 9.8% [6]. Oladeji and OlaOlorun discovered that 52.7% of infertile women in Nigeria suffer from depression [7]. According to a recent review of the literature on the prevalence of psychological disorders in infertility, between 25% and 60% of infertile people reported experiencing psychiatric symptoms, and their levels of anxiety and depression were noticeably higher than in fertile controls [8]. In Saudi Arabia, the prevalence of infertility among women is rising and has reached 18.9-23.3% at this time [9,10]. According to a 2011 study done in Saudi Arabia, 53.8% of infertile women reported having depression, compared to 37.2% of fertile women [11].

Despite the significant toll that infertility takes on mental health, comprehensive research specifically identifying the relevant mental health issues that are encountered by infertile Saudi Arabian women is lacking. To address these gaps in knowledge, our cross-sectional study aims to investigate issues like the prevalence rates, patterns, and severity levels of depression, anxiety, or stress for infertile women. This will help in the development of targeted interventions that will enhance the quality of life of these women. This cross-sectional study aims to measure the levels of depression, anxiety, and stress among infertile women in Saudi Arabia using the DASS-21 questionnaire (see Appendix) [12], which is a recommended method of measuring levels of depression, anxiety, or stress for an accurate assessment of the severity of their psychological disturbance. The objective is to compare and analyze the prevalence and severity of depression, anxiety, and stress among infertile and fertile women in Saudi Arabia.

## Materials and methods

Study design

This was an analytical cross-sectional study that targeted female visitors of governmental hospitals in Jeddah, Saudi Arabia. Jeddah, a city located in Western Saudi Arabia, has an estimated general population of around four million. We included outpatient visitors in the female waiting areas as part of this study.

Study population

All females of reproductive age were eligible to participate in the study. The study mainly included women with a confirmed diagnosis of primary or secondary infertility. Fertile women of the same age groups were also included for comparison purposes. Unmarried women, as well as those aged less than 18 or more than 50, were excluded from the study.

The sample size was calculated using an online tool (raosoft.com) A 5% margin of error, a 95% confidence level (CI), and a 50% distribution rate were used. The calculated sample size was 385. To include a comparison group of fertile women, the authors increased the sample size to 400, comprising half of the calculated sample size. A convenient sampling technique was utilized, inviting all visitors who met the eligibility criteria to participate in the study.

Data collection 

Data collection was conducted through a pre-structured questionnaire. Investigators were present at the waiting areas of outpatient clinics in the selected governmental hospitals. Each potential participant was approached and invited to take part in the study. Participants were provided with a link to the online questionnaire and asked to fill it out at their convenience. The assessment of mental illnesses was carried out using the DASS-21 [13].

Questionnaire

The questionnaire began by inquiring about demographic characteristics (e.g., age and gender). The DASS-21 was used as a self-report measure to assess the levels of depression, anxiety, and stress among participants in the study. The DASS-21, a widely used instrument, provided a reliable and valid assessment of these psychological constructs. The DASS-21 consisted of 21 items, with seven items dedicated to each of the three domains: depression, anxiety, and stress. Each item was rated on a Likert-type scale ranging from 0 to 3, indicating the frequency or severity of experienced symptoms over the past week. The response options included "Did not apply to me at all," "Applied to me to some degree, or some of the time," "Applied to me to a considerable degree, or a good part of the time," and "Applied to me very much, or most of the time." The Arabic DASS-21 was used in our study settings. 

Ethical consideration

Informed consent was obtained from participants who were provided comprehensive information about the study purpose, the nature of the questionnaire, and potential risks and benefits. Emphasis was placed on the voluntary nature of participation and the confidentiality of their responses. To guarantee participant privacy and anonymity, stringent measures were implemented. The data was securely stored, with access restricted to researchers involved in the study. The study received approval from the Ethics Committee of the Directorate of Health Affairs in Jeddah (approval number: A01826) ensuring compliance with ethical standards.

Statistical analysis

Data analysis was carried out using IBM SPSS Statistics, version 29.0 (released 2023, IBM Corp., Armonk, NY). Outcome variables were derived from the DASS-21, forming three continuous variables representing total scores for depression, anxiety, and stress. Subsequently, these outcome variables were categorized into five distinct groups: normal, mild, moderate, severe, and extremely severe for descriptive purposes.

Prior to performing descriptive analyses, we tested the continuous variables for normal distribution using Shapiro-Wilk and Kolmogorov-Smirnov tests. The results revealed a skewed data distribution, prompting us to summarize continuous variables using the median and interquartile range (IQR). Categorical variables, on the other hand, were summarized using frequency and proportions. To investigate the distinction between fertile and infertile women regarding depression, anxiety, and stress total scores, we utilized the Mann-Whitney U test. For demographic variables with more than two groups, the Kruskal-Wallis test was employed. Subgroup analysis was conducted to identify demographic associations with depression, anxiety, and stress specifically among infertile women. Comparisons of baseline characteristics for categorical variables were performed using the Chi-square test. Statistical significance was set at a level of less than 0.05.

## Results

In this cross-sectional study, a total of 397 women aged between 19 to 65 years (with a median age of 33 and an interquartile range from 29 to 40 years) were included. The majority of the sample were Saudi nationals (92.9%), married (89.9%), and held a bachelor's degree (69%). More than half of the women were unemployed (56.9%). The majority of the participants reported a monthly income of ≤5,000 SAR (41.6%), and most fell into the overweight BMI category (37.8%). Notably, 36.2% of the participants reported a diagnosis of infertility. Among the infertile women, 38.7% were married, in contrast to 61.3% of the non-diagnosed women, indicating a significant difference (p-value = 0.017). Despite this, there were no significant differences in nationality, educational level, employment status, monthly income, and BMI between infertile and fertile women. For more detailed demographic characteristics of the participants, refer to Table 1.

**Table 1:** Comparative analysis of the participants' demographic characteristics and their infertility diagnosis status *P-value calculated using the Fisher-Freeman-Halton exact test

Variables	Groups	Total	Have you been diagnosed with infertility before?		P-value
			Yes (%) n = 144	No (%) n = 253	
Nationality	Saudi	369 (92.9%)	138 (37.4%)	231 (62.6%)	0.090
	Non-Saudi	28 (7.1%)	6 (21.4%)	22 (78.6%)	
Marital status	Married	357 (89.9%)	138 (38.7%)	219 (61.3%)	0.008*
	Divorced	24 (6%)	5 (20.8%)	19 (79.2%)	
	Widowed	5 (1.3%)	1 (20%)	4 (80%)	
	Single	11 (2.8%)	0 (0%)	11 (100%)	
Educational level	Less than high school	11 (2.8%)	5 (45.5%)	6 (54.5%)	0.226
	High school	53 (13.4%)	20 (37.7%)	33 (62.3%)	
	Diploma	25 (6.3%)	14 (56%)	11 (44%)	
	Bachelor	274 (69%)	95 (34.7%)	179 (65.3%)	
	Higher education	34 (8.6%)	10 (29.4%)	24 (70.6%)	
Employment status	Employed	171 (43.1%)	66 (38.6%)	105 (61.4%)	0.402
	Unemployed	226 (56.9%)	78 (34.5%)	148 (65.5%)	
Monthly income (SAR)	≤5,000	165 (41.6%)	59 (35.8%)	106 (64.2%)	0.751
	5,000-10,000	106 (26.7%)	41 (38.7%)	65 (61.3%)	
	10,001-20,000	108 (27.2%)	36 (33.3%)	72 (66.7%)	
	>20,000	18 (4.5%)	8 (44.4%)	10 (55.6%)	
Body mass index (BMI)	Underweight	6 (1.5%)	1 (16.7%)	5 (83.3%)	0.140
	Normal	117 (29.5%)	47 (40.2%)	70 (59.8%)	
	Overweight	150 (37.8%)	60 (40%)	90 (60%)	
	Obese	124 (31.2%)	36 (29%)	88 (71%)	

**Figure 1:**
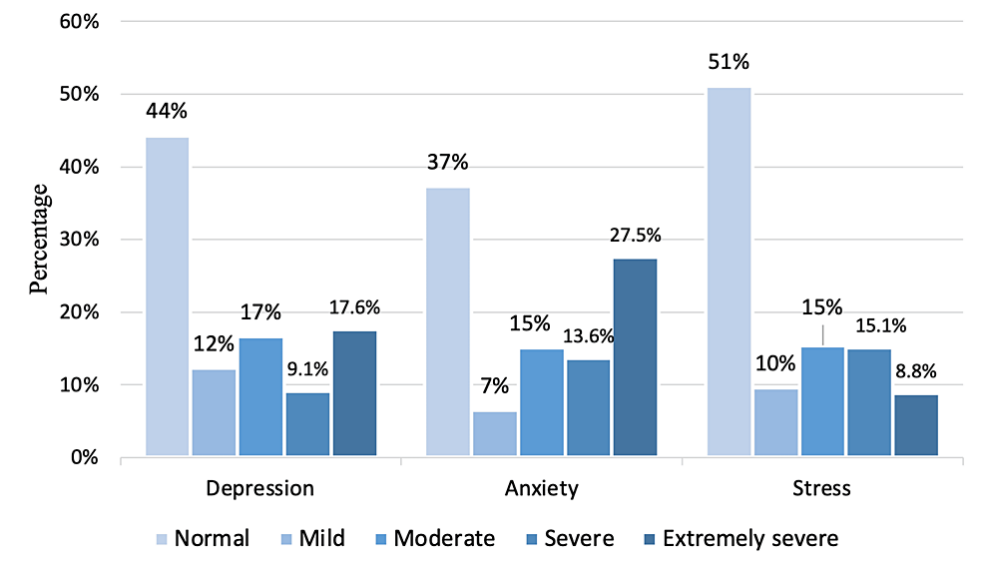
Distribution of the participants' severity levels for depression, anxiety, and stress

The median total score for depression was 12 with an interquartile range of 4-22. For anxiety, the median total score was 10, and the interquartile range was 4-20. The stress had a median total score of 14 with an interquartile range of 6-24. When examining the severity of these mental health conditions among the participants, the results were as follows: 44.3% of the participants demonstrated normal levels of depression, while 12.3% exhibited mild symptoms, 16.6% moderate, 9.1% severe, and 17.6% extremely severe symptoms. For anxiety, 37.3% of the participants were found to be within the normal range, whereas 6.5% presented mild symptoms, 15.1% moderate, 13.6% severe, and a substantial 27.5% extremely severe symptoms. Stress levels among the participants were normal for 51.1%, mild for 9.6%, moderate for 15.4%, severe for 15.1%, and extremely severe for 8.8%. These findings are visualized in detail in Figure 1.

**Figure 2:**
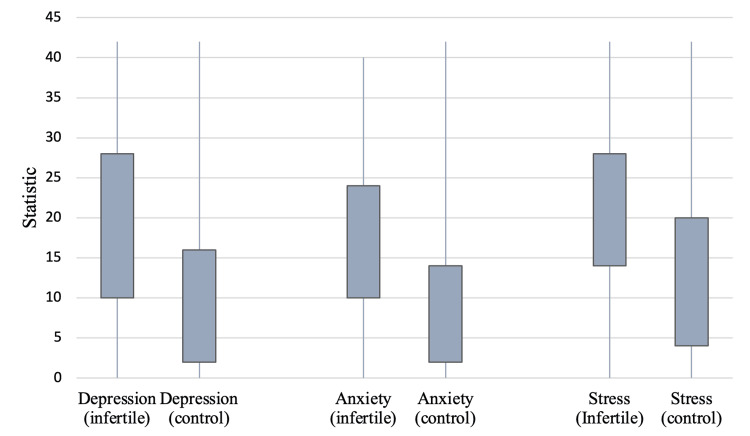
Demonstration of comparative median scores for depression, anxiety, and stress between infertile and fertile women

In our study, we observed noticeable differences in the median scores for depression, anxiety, and stress among infertile and fertile women. For infertile women, the median total score for depression was 18 with an interquartile range of 10-28. By contrast, for fertile women, the median total score for depression was 8 with an interquartile range of 2-16. Similarly, the median total score for anxiety was higher in infertile women (15, interquartile range of 10-24) compared to fertile women (8, interquartile range of 2-14). For stress, infertile women had a median total score of 20 with an interquartile range of 14-28, whereas fertile women had a median total score of 10 with an interquartile range of 4-20. The differences between the two groups were statistically significant for all three measures (p-value < 0.001 for each), which is further demonstrated in Figure 2.

Further analyses included the association between depression, anxiety, and stress with the demographic characteristics among infertile women, excluding the fertile control group. Our analysis revealed significant differences in depression, anxiety, and stress levels based on employment status and monthly income. Employed women reported higher median scores for depression (87), anxiety (84.5), and stress (84.5) compared to unemployed women, with P-values <0.001, 0.002, and 0.001, respectively. Moreover, women with a monthly income of 10,001-20,000 SAR had notably higher median scores for depression (89.56), anxiety (90.22), and stress (89.94) compared to other income groups, with P-values of 0.028, 0.019, and 0.02, respectively. However, nationality, marital status, educational level, and body mass index (BMI) showed no significant association with depression, anxiety, or stress levels among infertile women Table 2.

**Table 2:** Comparison of depression, anxiety, and stress scores across demographic characteristics among infertile women *P-value calculated using Mann-Whitney test. **P-value calculated using Kruskal-Wallis test.

Variables	Groups	Depression	Anxiety	Stress
		Mean rank		
Nationality	Saudi	71.84	72.77	71.82
	Non-Saudi	87.75	66.25	88.17
	P-value	0.359	0.707	0.346
Marital status	Married	72.41	71.94	72.27
	Divorced	84.8	97.4	88.2
	Widowed	23.5	25.5	26
	P-value	0.402	0.213	0.375
Educational level	Less than high school	70.9	74.2	68
	High school	78.75	79.63	69.35
	Diploma	69.64	60.07	61.32
	Bachelor	71.28	72.7	73.53
	Higher education	76.4	72.9	86.9
	P-value	0.952	0.764	0.656
Employment status	Employed	87	84.5	84.5
	Unemployed	60.2	62.4	62.3
	P-value	<0.001*	0.002*	0.001*
Monthly income (SAR)	≤5,000	64.06	65.19	62.42
	5,000-10,000	68.33	65.56	71.07
	10,001-20,000	89.56	90.22	89.94
	>20,000	79.38	82.19	75.69
	P-value	0.028**	0.019**	0.02**
Body mass index (BMI)	Underweight	54.5	123	60
	Normal	77.59	74.82	74.19
	Overweight	64.99	69.56	67.33
	Obese	78.88	72.97	79.25
	P-value	0.298	0.588	0.565

## Discussion

In this study, we discuss the impact of infertility on women, focusing on the psychological aspects of depression, anxiety, and stress using the DASS-21. Our study's population was composed of Saudi nationals. The population age in this study was about 33 years, well qualified but unemployed. Infertility in such a population is very challenging. In this study, psychological impact noted that infertile women had higher scores in depression, anxiety, and stress scales as compared to fertile women. This suggests that infertility can lead to intense emotional and psychological trauma. In societies where motherhood is considered a major aspect of a woman's identity, the inability to conceive raises feelings of hopelessness and anxiety [14]. In Saudi Arabia, where the family system is very developed, infertility can cause a social stigma. In fact, psychological impacts are not merely in a specific nation; this impact can be observed in different nationalities and considered a challenge among different socioeconomic and educational backgrounds. Employment status and increased psychological distress introduce a complex layer to this issue. Employed women may face more depression, anxiety, and stress and have pressure from their workplace and their family ​​[15,16].

For women with relatively higher socioeconomic status, the relationship between infertility and mental health is more complex. People with higher incomes report more depression, anxiety, and stress; this may reflect the high expectations and stress that come with managing certain economic activities in society [17]. Infertility and unemployment were found as risk factors for decreased mental health in our study. Another study has reported that unemployed females have a greater risk of stress than government employees [18]. This indeed can increase the burden of infertility among women significantly. Our data showed that the majority of participants experienced symptoms of depression, anxiety, and stress, particularly anxiety. Those intense symptoms can result from the uncertainty and impact of fertility treatment, which can be mentally draining and stressful. This is consistent with existing literature showing a relationship between pregnancy and increased stress, which called for mental health strategies to address increased cardiovascular risk [19]. Health management uses a variety of methods, including psychological assessment and support as an important part of the prenatal care package. There is a need to integrate mental health services to support infertile women. Meeting these needs should be done with continuous improvement in the research and care for women of reproductive age. This in turn can result in improvement in mental health and the outcome of infertility treatment [20].

Our study independently assessed the impact of educational attainment and employment status on depression, anxiety, and stress. The evidence suggests a potential confounding effect of these variables. A comparable case-control study conducted by Yusuf stratified women based on educational level and employment status, finding that the impact of infertility remained significant irrespective of these factors [21]. This evidence reinforces the view that infertility can be considered an independent risk factor for diminished mental health among women, regardless of their educational level or occupational status. While our study focused on the psychological implications of infertility, it is important to note that the adverse effects of infertility extend beyond mental health. For instance, in India, infertile women were found to have a lower quality of life in social, physical, and environmental aspects when compared to fertile women [22].

Strengths and limitations

The strengths of this study are multifold. For one, it includes a diverse sample size of 397 women ranging from the ages of 19 to 65. This wide age range allows for a comprehensive picture of the subject matter across different life stages. Second, the utilization of the DASS-21, a validated instrument for assessing mental health, lends credibility to the results of this study. Furthermore, the statistical analysis conducted was rigorous, adding a level of trustworthiness. However, this study's limitations lie in self-reported measures. This approach, while practical, may be subject to bias as participants could underreport or overreport certain elements. Furthermore, the cross-sectional nature of the study limits its ability to substantiate a cause-effect relationship between infertility and mental health. In addition, the study's geographical location in Jeddah, Saudi Arabia, could impose constraints on the generalizability of the findings. Cultural, social, and economic factors can vastly differ between regions, and these variations could affect the applicability of the study's conclusions to women in other locations.

## Conclusions

Our results revealed a significant association between infertility and increased levels of depression, anxiety, and stress among women in Saudi Arabia. The median scores for depression, anxiety, and stress were higher in infertile women compared to their fertile women. Specific demographic characteristics, such as employment status and monthly income, were found to be significantly related to mental health issues among infertile women. This suggests that socioeconomic factors may play a crucial role in the mental health outcomes of this population. These results emphasize the importance of including mental health support in infertility treatment plans. The high prevalence rates and severity levels of depression, anxiety, and stress indicate a pressing need for targeted interventions to improve the psychological well-being of infertile women. It is recommended that future research focus on developing and testing such interventions. Further studies could also explore the long-term effects of these mental health issues on the overall quality of life of infertile women.

## Appendices

DASS-21 questionnaire

Please read each statement and circle a number 0, 1, 2, or 3, which indicates how much the statement applied to you over the past week. There are no right or wrong answers. Do not spend too much time on any statement.

The rating scale is as follows: 0 Did not apply to me at all - NEVER 1 Applied to me to some degree, or some of the time - SOMETIMES 2 Applied to me to a considerable degree, or a good part of time - OFTEN 3 Applied to me very much, or most of the time - ALMOST ALWAYS

**Table 3:** Instructions and statements of the Depression Anxiety Stress Scale (DASS-21) [23].

Statement	0	1	2	3
I found it hard to wind down.				
I was aware of the dryness in my mouth.				
I couldn’t seem to experience any positive feelings at all.				
I experienced breathing difficulty (e.g., excessively rapid breathing, breathlessness in the absence of physical exertion).				
I found it difficult to work up the initiative to do things.				
I tended to overreact to situations.				
I experienced trembling (e.g., in my hands).				
I felt that I was using a lot of nervous energy.				
I was worried about situations in which I might panic and make a fool of myself.				
I felt that I had nothing to look forward to.				
I found myself getting agitated.				
I found it difficult to relax.				
I felt down-hearted and blue.				
I was intolerant of anything that kept me from getting on with what I was doing.				
I felt I was close to panic.				
I was unable to become enthusiastic about anything.				
I felt I wasn’t worth much as a person.				
I felt that I was rather touchy.				
I was aware of the action of my heart in the absence of physical exertion (e.g., sense of heart rate increase, heart missing a beat).				
I felt scared without any good reason.				
I felt that life was meaningless.				
